# The Learning Curve of Computer-Assisted Free Flap Jaw Reconstruction Surgery Using 3D-Printed Patient-Specific Plates: A Cumulative Sum Analysis

**DOI:** 10.3389/fonc.2021.737769

**Published:** 2021-09-16

**Authors:** Wang-yong Zhu, Wing Shan Choi, May Chun Mei Wong, Jingya Jane Pu, Wei-fa Yang, Yu-xiong Su

**Affiliations:** Division of Oral and Maxillofacial Surgery, Faculty of Dentistry, The University of Hong Kong, Hong Kong, SAR, China

**Keywords:** computer-assisted jaw reconstruction, virtual surgical planning, patient-specific surgical plate, three-dimensional printing technology, learning curve, cumulative sum analysis

## Abstract

**Background:**

Computer-assisted jaw reconstruction (CAJR) has benefits in reducing operation time and improving reconstruction accuracy, compared to conventional freehand jaw reconstruction. However, no information is available regarding learning curves in CAJR with the use of 3D-printed patient-specific surgical plates (PSSP). The purpose of this study was to assess surgical outcomes and learning curve for the first 58 consecutive CAJR using 3D-printed PSSP performed by a single surgical team in a single institution.

**Methods:**

In a prospective study, consecutive patients who underwent free flap CAJR using 3D-printed PSSP were included. The determination of proficiency, based on the cumulative sum of surgical success (no major adjustment of 3D-printed PSSP, flap survival) passing the acceptable boundary line of cumulative sum analysis, was the primary outcome. To find out any potential factors influencing the learning curve, baseline characteristics of patients were compared before and after proficiency achievement. Secondary outcomes included inflexion points of the total operation time, blood loss, length of hospital stay, and bone graft deviation, measured by the cumulative sum analysis.

**Results:**

From December 2016 to November 2020, 58 consecutive cases underwent surgery performed by a single surgical team. The overall surgical success rate was 94.8% (55/58). A three-stage learning curve of primary outcome was observed. The proficiency was achieved after 23 cases. The proportions of advanced tumor staging and concomitant surgery after obtaining proficiency were significantly higher than those before achieving proficiency (*p* = 0.046 and *p* < 0.001, respectively). Mean values of operation time, intraoperative blood loss, length of hospital stay, and bone graft deviation were 532.5 ± 119.2 min, 1,006.8 ± 547.2 ml, 16.1 ± 6.3 days, and 0.9 ± 1.2 mm, respectively. Two trends of learning curve were observed in the CUSUM analyses of total operation time, length of hospital stay, and bone graft deviation, in which the first and second inflexion points occurred between 8 and 17 cases and between 43 and 46 cases, respectively.

**Conclusion:**

Our results revealed a three-stage learning curve of CAJR with the use of PSSP, including initial learning, plateau, and overlearning. Based on CUSUM analysis, the surgical proficiency was achieved after 23 cases, and total operation time, length of hospital stay, and bone graft deviation stabilized after 8–17 cases.

## Introduction

The emerging technique of computer-assisted jaw reconstruction (CAJR), which facilitates preoperative surgery simulation and transfers the virtual plan to a real operation, significantly impacted conventional surgical approaches (1, 2). Studies have reported the benefits of CAJR compared to conventional freehand jaw reconstruction, including reductions of ischemia time, operation time, and related costs, and improvement of reconstruction accuracy (3–10).

With the increasing popularization of CAJR surgery, the needs of standardizing surgical training and optimizing patient outcomes are urgent. The cumulative sum (CUSUM) control chart is a sequential analysis technique in statistical quality control, typically used for monitoring change detection (11). Now, the concept of CUSUM analysis has been used by surgeons to assess the learning curve in complex surgeries, such as robot-assisted surgery and endoscopic surgery (12–14). It allows surgeons to precisely detect potential imperfections and then improve surgical outcomes. However, very sparse data are available on learning curves of free flap jaw reconstruction (15–18). To our best knowledge, there is no study reporting the learning curve in the practice of CAJR.

Our team started performing CAJR using 3D-printed patient-specific surgical plates (PSSP) in December 2016. We reported our first experience in 2018 and indicated that CAJR with PSSP is feasible, safe, and precise (19). This study aimed to analyze the surgical outcomes and learning curve for the first 58 consecutive CAJR cases using 3D-printed PSSP performed by a single surgical team in a single institution.

## Materials and Methods

This study was approved by the Institutional Review Board of the University of Hong Kong/Hospital Authority Hong Kong West Cluster (No. UW 16-315), registered in ClinicalTrials.gov with a No. of NCT03057223 and in The University of Hong Kong Clinical Trials Centre with a study identifier of HKUCTR-2113 (www.HKUCTR.com).

### Surgical Interventions

From December 2016 to November 2020, all consecutive patients who underwent CAJR with 3D-printed PSSP performed by a single surgical team led by the same chief surgeon in the Queen Mary Hospital in Hong Kong were enrolled without dropout. Before December 2016, the surgical team had no previous experience in using PSSP.

The virtual planning, design, and fabrication of 3D-printed PSSP and surgical techniques have been described in our previous publications (19, 20). Patients indicated for jaw surgery were arranged to undertake contrast-enhanced CT scan of head and neck and the donor site. The virtual surgical planning was done using Proplan CMF 3.0 software (Materialise, Leuven, Belgium). To exactly transfer the surgical plan to the operation room, we designed PSSP in line with the planned surgery using the 3-Matic software (Materialise, Leuven, Belgium). The surgical templates were printed using the stereolithography technology from high-strength resin. The plates were printed using Grade 2 pure titanium by selective laser melting technology. All the surgical procedures and perioperative management were conducted in a routine manner, except that osteotomies, bone movements, and flap inset were guided by the prepared 3D-printed patient-specific surgical templates and fixed by the 3D-printed patient-specific pure titanium plate. Patients were followed up regularly. Plain x-ray image and CT/CBCT scanning were done approximately 1 month after surgery.

### Outcomes

The primary outcome was the achievement of proficiency. All CAJR cases were recorded chronologically by operation date. Proficiency was based on the cumulative sum of surgical success reaching the acceptable boundary line of the CUSUM analysis. Surgical success was defined as no change of 3D-printed PSSP (major adjustment of plates, including the need for bending plate and conversion to conventional commercialized titanium plates) and flap survival. Cases were divided into two groups according to the achievement of proficiency, and the baseline characteristics were compared to analyze the potential factors influencing the learning curves.

The secondary outcomes were the stabilization of total operation time, intraoperative blood loss, length of hospital stay, and reconstruction accuracy to a steady state. The determination of stabilization was based on the inflection point of the cumulative graph. The accuracy of jaw reconstruction 1 month postoperatively was measured by calculating the deviation distance between preoperative planned and postoperative achieved bone graft positions using the same method described previously (4).

### CUSUM Analysis

CUSUM analysis was performed to detect subtle deviations of the surgeon’s performance in primary and secondary outcomes (21).

The overall fibula flap failure rate was revealed as 7.0% (*n* = 161/2,305) by the latest systematic review and meta-analysis (22). Since fibula flaps were used in most of our cases, we utilized 7.0% as the current failure rate of free flap. We reviewed the available studies using PSSP in maxillofacial reconstruction (**Table 1**) and pooled the current failure rate of PSSP as 4.4% (*n* = 6/136) (19, 23–27). Therefore, the overall success rate of implementing free flap and PSSP was 88.9%, and the acceptable level of surgical failure (p0) was set at 11.1% [100% − (100% − 7.0%) × (100% − 4.4%)] in the CUSUM analysis. A chosen level of surgical failure rate (p1) reflecting a change in surgical performance was set as two times the acceptable level of surgical failure (28). All calculation procedures and intermediate values are shown in **Appendix**. Briefly, with each surgical success obtained, the line would rise by 0.162; with each failure, the line would fall by 0.838. Type 1 and type 2 errors, the probabilities of wrongly accusing the surgeon of unacceptable performance and acceptable performance, were set as 0.10, which were considered rational (28). Boundary lines were calculated to determine whether the surgical performance was acceptable (H_1_) or unacceptable (H_0_). Once the line reached H_1_ or H_0_, proficiency was obtained or lost, respectively.

**Table 1:** Literature review on studies using PSSP in maxillofacial reconstruction.

Author	Total No. of Cases Using PSSP	Failed No. of Cases Using PSSP
Frank Wilde, 2015 (23)	32	6
Majeed Rana, 2017 (24)	22	0
Yang, 2018 (19)	10	0
David Öhman, 2019 (25)	5	0
Philipp Jehn, 2020 (26)	20	0
Zavattero, 2021 (27)	47	0

The sequential differences of total operation time, intraoperative blood loss, length of hospital stay, and bone graft deviation between each case and the mean value were also detected by CUSUM (29). The mean values of each variable were calculated and used as the reference. Briefly, with each variable more or less than the mean value, the line would rise or fall by the absolute difference, respectively. The best-fit curve and its corresponding equation were determined with the cftool command in Excel (version 2019; Microsoft Corporation, USA).

### Statistical Analysis

All statistics were calculated using SPSS Statistics (version 26.0; IBM Corporation, Chicago, IL, USA). Data were presented as number (percentage) for categorical data and mean ± standard deviation for continuous data. Independent samples *t* test was used to detect differences in the means in the patients’ age and donor bone length. Chi-square test was used to detect differences in the proportions of gender, diagnosis, TNM classification, surgical site, bone segments, and concomitant surgery. *p* < 0.05 was considered statistically significant.

## Results

A total of 58 consecutive patients who underwent CAJR using PSSP were included. Baseline characteristics of total cases are shown in **Table 2**.

**Table 2:** Baseline characteristics and surgical details.

Characteristics	Total cases (*n* = 58)
Gender (*n*, %)	
Male	26 (44.8%)
Female	32 (55.2%)
Age (years)	59.3 ± 16.0
Diagnosis (*n*, %)	
Malignant tumor	40 (69.0%)
Benign tumor	13 (22.4%)
Others^a^	5 (8.6%)
TNM classification^b^ (40 cases) (*n*, %)	
Stage I and II	15 (37.5%)
Stage III and IV	25 (62.5%)
Surgical site (*n*, %)	
Mandible	44 (75.9%)
Maxilla	14 (24.1%)
Donor bone graft (*n*, %)	
Fibula	54 (93.1%)
Iliac crest	4 (6.9%)
Donor bone length (mm)	89.7 ± 31.0
Bone segments (*n*, %)	
One	12 (20.7%)
Two	31 (53.4%)
Three and more	15 (25.9%)
Concomitant surgery (*n*, %)	
None	36 (62.1%)
Simultaneous dental implant	21 (36.2%)
Radial forearm flap	1 (1.7%)

a Others: osteoradionecrosis of the jaw (n = 4) and mandibular defect secondary to malignancy resection (n = 1).

b According to the AJCC (American Joint Committee on Cancer) Cancer Staging Manual (8th Edition).

### Primary Outcome

**Figure 1:**
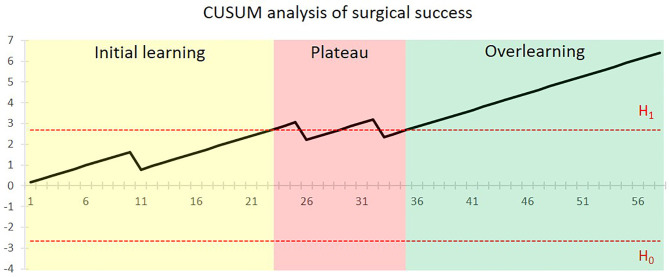
CUSUM analysis of surgical success for the CAJR with PSSP performed by the surgical team. The *x*-axis shows the chronological sequence of cases. The black line indicates the cumulative sum of surgical success. The horizontal red dotted lines represent the acceptable boundary line H_1_ and unacceptable boundary line H_0_. Proficiency was first achieved after 35 cases.

Altogether, surgical failure occurred in 5.2% of patients (3/58). One was intraoperative flap failure due to sclerotic vessels and recurrent arterial thrombosis. The other two cases were postoperative flap failures caused by venous compromise of flap at postoperative day 4 and late-stage artery thrombosis at postoperative day 10. 3D-printed PSSP were successfully used in all the patients. **Figure 1** shows the CUSUM analysis of the surgical success for the surgeon. After a surgical failure occurred in the 11th case, a steadily climbing line was seen and proficiency was first obtained after 23 cases. A new concomitant surgery of simultaneous dental implant placement with or without immediate loading was added to selective cases from the 23rd case. Two surgical failures occurred in the 26th and 33rd cases and the surgical performance line dropped below the acceptable boundary line. After that, proficiency was completely regained after 35 cases.

According to the achievement of proficiency, we divided the patients into two groups. From the baseline characteristics of the two groups in **Table 3**, there was no significant difference in the patient’s baseline features, except TNM stage (*p* = 0.046) and concomitant surgery (*p* < 0.001). From the 24th to 58th cases, the proportions of stage III & IV malignancy and concomitant surgery were significantly higher than the first 23 cases, indicating an increased proportion of cases with advanced stage malignancy and more complex surgery after first achieving the proficiency.

**Table 3:** Comparison of baseline characteristics among chronological 2 groups.

Characteristics	Group 1 (Case No.1-23)	Group 2 (Case No.24-58)	P value
Gender (n; %)			
Male	8 (34.8%)	18 (51.4%)	0.212
Female	15 (65.2%)	17 (48.6%)	
Age (years)	58.7±15.0	59.6±16.9	0.831
Diagnosis (n; %)			
Malignant tumor	16 (69.6%)	24 (68.6%)	0.523
Benign tumor	4 (17.4%)	9 (25.7%)	
Others	3 (13.0%)	2 (5.7%)	
TNM classification (40 cases) (n; %)			
Stage I & II	9 (56.3%)	6 (25.0%)	0.046
Stage III & IV	7 (43.8%)	18 (75.0%)	
Surgical site (n; %)			
Mandible	17 (73.9%)	27 (77.1%)	0.779
Maxilla	6 (26.1%)	8 (22.9%)	
Donor bone graft (n; %)			
Fibula	21 (91.3%)	33 (94.3%)	0.522
Iliac crest	2 (8.7%)	2 (5.7%)	
Donor bone length (mm)	89.6±36.5	89.7±27.4	0.982
Bone segments (n; %)			
One	4 (17.4%)	8 (22.9%)	0.771
Two	12 (52.2%)	19 (54.3%)	
Three and more	7 (30.4%)	8 (22.9%)	
Concomitant surgery (n; %)			
No	22 (95.7%)	14 (40.0%)	<0.001
Yes	1 (4.3%)	21 (60.0%)	

### Secondary Outcomes

**Figure 2:**
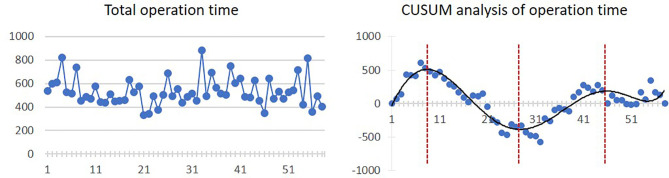
Linear graph and CUSUM analysis of total operation time in CAJR applying patient-specific surgical plates. The black line represents the curve of best fit in a general model with equation, *y* = 4E−06*x* ^6^ − 0.0006*x* ^5^ + 0.0299*x* ^4^ − 0.3358*x* ^3^ − 9.1732*x* ^2^ + 168.85*x* − 187.47, *R*² = 0.8506.

The mean operation time was 532.5 ± 119.2 min. The inflexion point is the 8th case, from which the operation time started to diminish, although there was a slight trend of increasing operation time from the 28th case to the 46th case. The linear and CUSUM analysis graphs of total operation time are shown in **Figure 2**.

**Figure 3:**
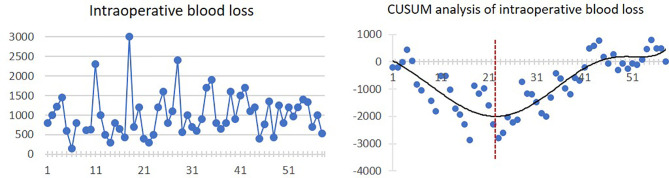
Linear graph and CUSUM analysis of intraoperative blood loss in CAJR applying patient-specific surgical plates. The black line represents the curve of best fit in a general model with equation, *y* = 4E−06*x* ^6^ − 0.0006*x* ^5^ + 0.0274*x* ^4^ − 0.2982*x* ^3^ − 1.1805*x* ^2^ − 94.942*x* + 133.67, *R*² = 0.7233.

Mean intraoperative blood loss was 1,006.8 ± 547.2 ml. There was a trend of decreasing blood loss in the first 22 cases. After that, the intraoperative blood loss was increasing. The linear and CUSUM analysis graphs of intraoperative blood loss are shown in **Figure 3**.

**Figure 4:**
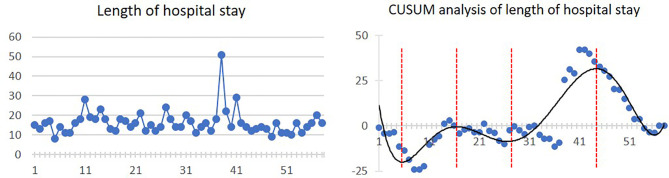
Linear graph and CUSUM analysis of hospital stay length in CAJR applying patient-specific surgical plates. The black line represents the curve of best fit in a general model with equation, *y* = 6E−07*x* ^6^ − 0.0001*x* ^5^ + 0.0077*x* ^4^ − 0.2461*x* ^3^ + 3.7349*x* ^2^ − 23.636*x* + 31.376, *R*² = 0.7912.

Mean length of hospital stay was 16.1 ± 6.3 days. Four inflexion points were presented at the 5th, 17th, 28th, and 45th cases, respectively. The linear and CUSUM analysis graphs of hospital stay length are shown in **Figure 4**.

**Figure 5:**
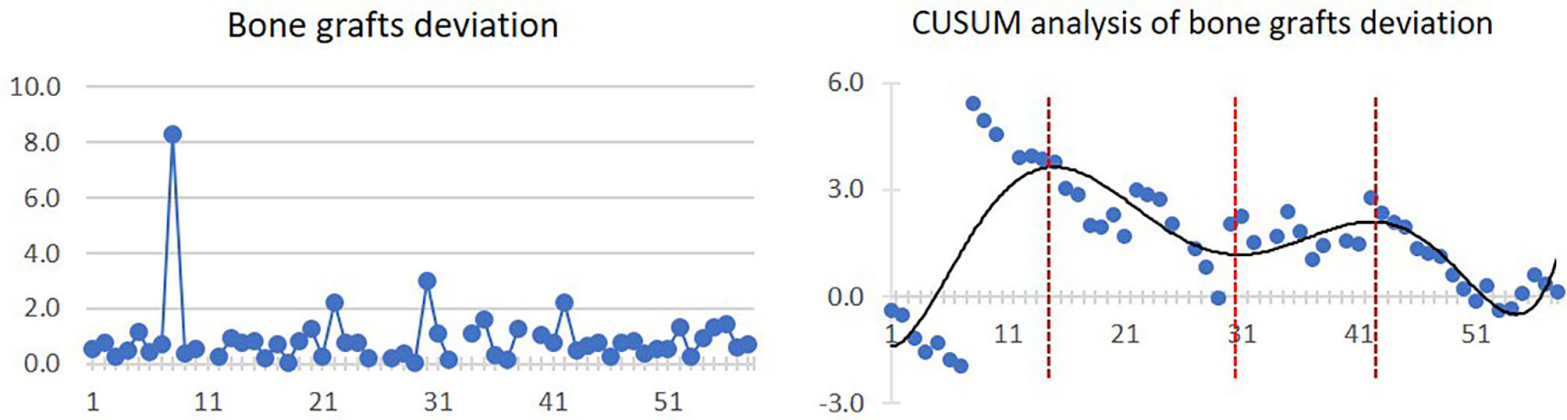
Linear graph and CUSUM analysis of bone grafts deviation in CAJR applying patient-specific surgical plates. The black line represents the curve of best fit in a general model with equation, *y* = 5E−08*x* ^6^ − 8E−06*x* ^5^ + 0.0005*x* ^4^ − 0.0148*x* ^3^ + 0.1689*x* ^2^ − 0.2517*x* − 1.307, *R*² = 0.5936.

Mean bone graft deviation was 0.9 ± 1.2 mm. After 15 cases, the bone graft deviation started to diminish as a general trend. However, from the 31st to 43rd cases, a slightly increasing trend of bone graft deviation was observed. The linear and CUSUM analysis graphs of bone grafts deviation are shown in **Figure 5**.

## Discussion

The concept of learning curve in medicine was commonly defined as the time taken and/or the number of procedures an average surgeon needs to be able to perform a procedure independently with a reasonable outcome (30). As modern surgical training is always beset by problems like increased working hours, inadequate training facilities, lack of resources, and medicolegal issues (31), the understanding of learning curves on surgical procedures can make the training more efficient and standard. However, a study reviewed assessments of learning curve in health technologies and indicated that learning curves were rarely evaluated formally with a proper study design and statistical method (32). As a new tool, the CUSUM technique for learning curve has been introduced and proposed as a useful instrument in the field of surgical training, which allows quantitative monitoring of individual performance during the learning process (28, 33).

Nowadays, computer-assisted surgery is increasingly utilized in reconstructive surgery, while no literature has reported the learning curve for this surgical procedure. In the present study, we performed CUSUM analysis on the learning curve of CAJR using 3D-printed PSSP by a single surgical team. Although operation time is the most common determinant for the learning curve on surgery, the assessment on the basis of a single parameter might be simplistic (12, 34). We tried to analyze trends in multidimensional variables in the present study.

For the primary outcome, our study revealed a three-stage learning pattern of CAJR with the use of PSSP, including initial learning, plateau, and overlearning. The first stage is initial learning. The learning curve regarding surgical success stabilized after 11 cases, and first achieved proficiency after 23 cases. The second stage is plateau. When surgeons felt competent in this surgical procedure, the significantly increased proportions of tumor staging and concomitant surgery suggested that the complexity of surgery after 23 cases was higher than the earlier cases. The concomitant surgery, simultaneous dental implant placement with the aid of a “three-in-one” patient-specific surgical guide (35), was also a technically challenging procedure. Accordingly, a fluctuation of proficiency was observed at this stage. The final stage is overlearning. A steady proficiency was completely achieved after 35 cases. Overall, the surgical failures in our cohort were infrequent (5.2%), and no performance of the surgeon below the unacceptable boundary line was observed, which suggests that application of 3D-printed PSSP in CAJR with free flap is safe and feasible.

For the secondary outcomes, we found that although the four graphs of CUSUM analysis had different curve shapes, a similar ascending trend occurred from 23–31 cases to 43–46 cases. The increased complexity after 23 cases could also reasonably explain the increased total operation time and intraoperative blood loss. As a result, we actually observed two phenomena of “learning curve” in the CUSUM analysis graphs of the total operation time, length of hospital stay, and bone graft deviation. In the first learning curve (from 1st to 28th–31st cases), the inflexion points occurred between 8 and 17 cases, from which operation time, length of hospital stay, and bone graft deviation started to diminish. In the second learning curve (from 28th–31st to 58th cases), stabilizations of the operation time, length of hospital stay, and bone graft deviation occurred between 43 and 46 cases.

The main novelty of this study was using the CUSUM technique to analyze the learning curve of CAJR with PSSP. We utilized a chosen level of surgical failure rate as strict as two times the currently acceptable level (11.1%) to determine proficiency, which was one strength of our study (28). The cohort of patients from a prospective clinical trial without dropout was also the strength. Since no study described the learning curve of CAJR surgery, our work can provide the first quantitative assessment on this topic to the literature. It should be noted that our surgical team had previous experience in conventional free flap jaw reconstruction surgery and also CAJR without the use of PSSP (36), which could help shorten the learning process. Learning curve varies from different surgeons, but its stage pattern may be similar. Among three stages, the occurrence and persistence of a plateau depend on many reasons, such as the interference by previous experience, the nature of the task, and the motivation (37). Finding out the correct cause and getting over this period will be an important portion in surgical training.

There are certain limitations of the present study that need to be addressed. First, the CUSUM analysis included different diseases (malignant and benign tumors), surgeries (maxillary and mandibular reconstructions), and free flaps (fibular and iliac flaps), which influenced the homogeneity of enrolled cases. A multi-center clinical trial, with a big enough sample size of the same disease and surgery, might be needed to overcome this limitation. However, it will lead to other limitations of different expertise of multiple surgical teams and hospital setting. Second, post-operative oral function and quality of life are important outcomes for jaw reconstruction. However, there are a lot of confounding factors influencing these outcomes, which will need a well-designed prospective randomized control trial for further investigation. Thus, we did not include them in the present analysis. Last, the learning curve of time spent on preoperative preparation, such as virtual surgical planning and PSSP design, was not reported in this study. Previously, we reported that the time spent on virtual surgery and plate design was 18.8 ± 13.2 h, and the time taken for 3D printing, post-processing, and product delivery was 162.9 ± 55.2 h (38). The consecutive data of the time spent on preoperative preparation with a large sample size would provide a better understanding of the whole learning process of CAJR using PSSP.

## Conclusion

A three-stage learning pattern of CAJR with the use of PSSP was revealed, including initial learning, plateau, and overlearning, which may guide the clinical teaching and training of CAJR using PSSP. Based on CUSUM analysis, surgical proficiency was obtained after 23 cases. Stabilization of total operation time, length of hospital stay, and bone graft deviation occurred after 8–17 cases. Our study provided evidence to guide the training of this new surgical procedure to ensure patient safety and clinical outcomes.

## Data Availability Statement

The raw data supporting the conclusions of this article will be made available by the authors, without undue reservation.

## Ethics Statement

The studies involving human participants were reviewed and approved by the Institutional Review Board of the University of Hong Kong/Hospital Authority Hong Kong West Cluster. The patients/participants provided their written informed consent to participate in this study.

## Author Contributions

W-yZ and Y-xS: study design. Y-xS: study supervision. W-yZ, WC, JP and W-fY: data collection. W-yZ and MW: manuscript preparation, statistical analysis, and data interpretation. All authors contributed to the article and approved the submitted version.

## Funding

The study was funded by the Health and Medical Research Fund (No. 08192096), Hong Kong SAR, Guangdong Science and Technology Department (No. 2019A050516001), University Research Committee Platform Technology Funding 2021, and HKU Seed Fund for Translational and Applied Research (202010160032).

## Conflict of Interest

The authors declare that the research was conducted in the absence of any commercial or financial relationships that could be construed as a potential conflict of interest.

## Publisher’s Note

All claims expressed in this article are solely those of the authors and do not necessarily represent those of their affiliated organizations, or those of the publisher, the editors and the reviewers. Any product that may be evaluated in this article, or claim that may be made by its manufacturer, is not guaranteed or endorsed by the publisher.

## Appendix

Current acceptable failure rate (p0)=0.111

Chosen level of failure rate (p1)=0.222

Type 1 error (α)=0.1

Type 2 error (β)=0.1

P=ln(p1/p0)=ln(0.222/0.111)=ln2 = 0.690

Q=ln[(1-p0)/(1-p1)]=ln[0.889/0.778]=ln1.143 = 0.133

a=In[(1-β)/α]=In(0.9/0.1)=2.197

b=In[(1-α)/β]=In(0.9/0.1)=2.197

s=Q/(P+Q)=0.133/(0.690 + 0.133)=0.162 (With success, graph goes 0.162 upwards, and with failure, graph goes 0.838 [1-s] downwards.)

Unacceptable boundary line: H_0_=-b/(P+Q)=-2.197/(0.690 + 0.133)=-2.670

Acceptable boundary line: H_1_=a/(P+Q)=2.197/(0.690 + 0.133)=2.670

## References

[1] Okay D Al Shetawi AH Moubayed SP Mourad M Buchbinder D Urken ML. Worldwide 10-Year Systematic Review of Treatment Trends in Fibula Free Flap for Mandibular Reconstruction. J Oral Maxillofac Surg (2016) 74(12):2526–31. doi: 10.1016/j.joms.2016.06.170 27400143

[2] Kakarala K Shnayder Y Tsue TT Girod DA. Mandibular Reconstruction. Oral Oncol (2018) 77:111–7. doi: 10.1016/j.oraloncology.2017.12.020 29362116

[3] Powcharoen W Yang WF Yan Li K Zhu W Su YX. Computer-Assisted Versus Conventional Freehand Mandibular Reconstruction With Fibula Free Flap: A Systematic Review and Meta-Analysis. Plast Reconstr Surg (2019) 144(6):1417–28. doi: 10.1097/prs.0000000000006261 31764662

[4] Yang WF Choi WS Wong MC Powcharoen W Zhu WY Tsoi JK. Three-Dimensionally Printed Patient-Specific Surgical Plates Increase Accuracy of Oncologic Head and Neck Reconstruction Versus Conventional Surgical Plates: A Comparative Study. Ann Surg Oncol (2021) 28(1):363–75. doi: 10.1245/s10434-020-08732-y PMC7752789 32572853

[5] Pucci R Weyh A Smotherman C Valentini V Bunnell A Fernandes R. Accuracy of Virtual Planned Surgery Versus Conventional Free-Hand Surgery for Reconstruction of the Mandible With Osteocutaneous Free Flaps. Int J Oral Maxillofac Surg (2020) 49(9):1153–61. doi: 10.1016/j.ijom.2020.02.018 32197824

[6] Weitz J Bauer FJM Hapfelmeier A Rohleder NH Wolff KD Kesting MR. Accuracy of Mandibular Reconstruction by Three-Dimensional Guided Vascularised Fibular Free Flap After Segmental Mandibulectomy. Br J Oral Maxillofac Surg (2016) 54(5):506–10. doi: 10.1016/j.bjoms.2016.01.029 26898519

[7] Bao T He J Yu C Zhao W Lin Y Wang H. Utilization of a Pre-Bent Plate-Positioning Surgical Guide System in Precise Mandibular Reconstruction With a Free Fibula Flap. Oral Oncol (2017) 75:133–9. doi: 10.1016/j.oraloncology.2017.11.011 29224810

[8] De Maesschalck T Courvoisier DS Scolozzi P. Computer-Assisted Versus Traditional Freehand Technique in Fibular Free Flap Mandibular Reconstruction: A Morphological Comparative Study. Eur Arch Otorhinol (2017) 274(1):517–26. doi: 10.1007/s00405-016-4246-4 27501991

[9] Ciocca L Marchetti C Mazzoni S Baldissara P Gatto MRA Cipriani R. Accuracy of Fibular Sectioning and Insertion Into a Rapid-Prototyped Bone Plate, for Mandibular Reconstruction Using CAD-CAM Technology. J Cranio-Maxillo-Facial Surg (2015) 43(1):28–33. doi: 10.1016/j.jcms.2014.10.005 25434288

[10] Zhang L Liu Z Li B Yu H Shen SG Wang X. Evaluation of Computer-Assisted Mandibular Reconstruction With Vascularized Fibular Flap Compared to Conventional Surgery. Oral Surg Oral Med Oral Pathol Oral Radiol (2016) 121(2):139–48. doi: 10.1016/j.oooo.2015.10.005 26792754

[11] Grigg OA Farewell VT Spiegelhalter DJ. Use of Risk-Adjusted CUSUM and RSPRT Charts for Monitoring in Medical Contexts. Stat Methods Med Res (2003) 12(2):147–70. doi: 10.1177/096228020301200205 12665208

[12] Barrie J Jayne DG Wright J Murray CJC Collinson FJ Pavitt SH. Attaining Surgical Competency and Its Implications in Surgical Clinical Trial Design: A Systematic Review of the Learning Curve in Laparoscopic and Robot-Assisted Laparoscopic Colorectal Cancer Surgery. Ann Surg Oncol (2014) 21(3):829–40. doi: 10.1245/s10434-013-3348-0 24217787

[13] Song X Wang D Sun X Wang J Liu Z Liu Q. Cumulative Sum Analysis of the Learning Curve for Endoscopic Resection of Juvenile Nasopharyngeal Angiofibroma. Surg Endosc (2018) 32(7):3181–91. doi: 10.1007/s00464-018-6035-1 29368283

[14] Antoun A Chau J Alsharqawi N Kaneva P Feldman LS Mueller CL. P338: Summarizing Measures of Proficiency in Transanal Total Mesorectal Excision-A Systematic Review. Surg Endosc (2021) 35(8):4817–24. doi: 10.1007/s00464-020-07935-4 32875417

[15] Kim H Jeong WJ Ahn SH. Results of Free Flap Reconstruction After Ablative Surgery in the Head and Neck. Clin Exp Otorhinolaryngol (2015) 8(2):167–73. doi: 10.3342/ceo.2015.8.2.167 PMC4451543 26045917

[16] Ratnagiri R Jena S Parvathi P Srikanth R Raju GSN. Reconstruction in Head-and-Neck Cancers - Analysis of the Learning Curve. Natl J Maxillofac Surg (2018) 9(2):191–5. doi: 10.4103/njms.NJMS_66_17 PMC6251285 30546234

[17] Cariati P Cabello Serrano A Monsalve Iglesias F Roman Ramos M Fernandez Solis J Martinez Lara I. Unfavorable Outcomes in Microsurgery: Possibilities for Improvement. J Plast Surg Handb Surg (2019) 53(5):279–87. doi: 10.1080/2000656X.2019.1606005 31066601

[18] Fliss E Yanko R Bracha G Teman R Amir A Horowitz G. The Evolution of the Free Fibula Flap for Head and Neck Reconstruction: 21 Years of Experience With 128 Flaps. J Reconstr Microsurg (2021) 37(4):372–9. doi: 10.1055/s-0040-1717101 32998171

[19] Yang WF Choi WS Leung YY Curtin JP Du R Zhang CY. Three-Dimensional Printing of Patient-Specific Surgical Plates in Head and Neck Reconstruction: A Prospective Pilot Study. Oral Oncol (2018) 78:31–6. doi: 10.1016/j.oraloncology.2018.01.005 29496055

[20] Pu JJ Choi WS Yu P Wong MCM Lo AWI Su YX. Do Predetermined Surgical Margins Compromise Oncological Safety in Computer-Assisted Head and Neck Reconstruction? Oral Oncol (2020) 111:104914. doi: 10.1016/j.oraloncology.2020.104914 32712577

[21] Steiner SH Cook RJ Farewell VT Treasure T. Monitoring Surgical Performance Using Risk-Adjusted Cumulative Sum Charts. Biostatistics (2000) 1(4):441–52. doi: 10.1093/biostatistics/1.4.441 12933566

[22] Awad ME Altman A Elrefai R Shipman P Looney S Elsalanty M. The Use of Vascularized Fibula Flap in Mandibular Reconstruction; A Comprehensive Systematic Review and Meta-Analysis of the Observational Studies. J Cranio-Maxillo-Facial Surg (2019) 47(4):629–41. doi: 10.1016/j.jcms.2019.01.037 30782453

[23] Wilde F Hanken H Probst F Schramm A Heiland M Cornelius C-P. Multicenter Study on the Use of Patient-Specific CAD/CAM Reconstruction Plates for Mandibular Reconstruction. Int J Comput Assist Radiol Surg (2015) 10(12):2035–51. doi: 10.1007/s11548-015-1193-2 25843949

[24] Rana M Chin S-J Muecke T Kesting M Groebe A Riecke B. Increasing the Accuracy of Mandibular Reconstruction With Free Fibula Flaps Using Functionalized Selective Laser-Melted Patient-Specific Implants: A Retrospective Multicenter Analysis. J Cranio-Maxillofac Surg (2017) 45(8):1212–9. doi: 10.1016/j.jcms.2017.04.003 28552201

[25] Öhman D Schaefer C Nannmark U Kjeller G Malmström J. Mandible Reconstruction With Patient-Specific Implants: Case Report of Five Consecutive Patients. Int J Oral Maxillofac Implants (2019) 34(1):e7–e11. doi: 10.11607/jomi.6913 30521658

[26] Jehn P Spalthoff S Korn P Zeller A-N Dittmann J Zimmerer R. Patient-Specific Implant Modification for Alloplastic Bridging of Mandibular Segmental Defects in Head and Neck Surgery. J Cranio-Maxillofac Surg (2020) 48(3):315–22. doi: 10.1016/j.jcms.2020.01.018 32089430

[27] Zavattero E Bolzoni A Dell'Aversana G Santagata M Massarelli O Ferri A. Accuracy of Fibula Reconstruction Using Patient-Specific Cad/Cam Plates: A Multicenter Study on 47 Patients. Laryngoscope (2021) 131(7):E2169–75. doi: 10.1002/lary.29379 33452834

[28] Bolsin S Colson M. The Use of the Cusum Technique in the Assessment of Trainee Competence in New Procedures. Int J Qual Health Care (2000) 12(5):433–8. doi: 10.1093/intqhc/12.5.433 11079224

[29] Kim CW Kim WR Kim HY Kang J Hur H Min BS. Learning Curve for Single-Incision Laparoscopic Anterior Resection for Sigmoid Colon Cancer. J Am Coll Surg (2015) 221(2):397–403. doi: 10.1016/j.jamcollsurg.2015.02.016 26070396

[30] Subramonian K Muir G. The 'Learning Curve' in Surgery: What Is it, How do We Measure It and Can We Influence It? BJU Int (2004) 93(9):1173–4. doi: 10.1111/j.1464-410X.2004.04891.x 15180598

[31] Chikwe J de Souza AC Pepper JR. No Time to Train the Surgeons. BMJ (Clinical Res ed) (2004) 328(7437):418–9. doi: 10.1136/bmj.328.7437.418 PMC344249 14976074

[32] Ramsay CR Grant AM Wallace SA Garthwaite PH Monk AF Russell IT. Statistical Assessment of the Learning Curves of Health Technologies. Health Technol Assess (2001) 5(12):1–79. doi: 10.3310/hta5120 11319991

[33] Biau DJ Williams SM Schlup MM Nizard RS Porcher R. Quantitative and Individualized Assessment of the Learning Curve Using LC-CUSUM. Br J Surg (2008) 95(7):925–9. doi: 10.1002/bjs.6056 18498126

[34] Pernar LIM Robertson FC Tavakkoli A Sheu EG Brooks DC Smink DS. An Appraisal of the Learning Curve in Robotic General Surgery. Surg Endosc (2017) 31(11):4583–96. doi: 10.1007/s00464-017-5520-2 28411345

[35] Zhu WY Su YX Pow EHN Yang WF Qin L Choi WS. “Three-In-One” Patient-Specific Surgical Guides for Simultaneous Dental Implants in Fibula Flap Jaw Reconstruction: A Prospective Case Series. Clin Implant Dent Relat Res (2021) 23(1):43–53. doi: 10.1111/cid.12954 33180980

[36] Zheng G-S Su Y-X Liao G-Q Liu H-C Zhang S-E Liang L-Z. Mandibular Reconstruction Assisted by Preoperative Simulation and Accurate Transferring Templates: Preliminary Report of Clinical Application. J Oral Maxillofac Surg (2013) 71(9):1613–8. doi: 10.1016/j.joms.2013.02.018 23810619

[37] N Pam MS. LEARNING PLATEAU. PsychologyDictionary.org (2018). Available at: https://psychologydictionary.org/learning-plateau/ (Accessed September 9, 2021).

[38] Yang WF Zhang CY Choi WS Zhu WY Li DTS Chen XS. A Novel 'Surgeon-Dominated' Approach to the Design of 3D-Printed Patient-Specific Surgical Plates in Mandibular Reconstruction: A Proof-of-Concept Study. Int J Oral Maxillofac Surg (2020) 49(1):13–21. doi: 10.1016/j.ijom.2019.05.005 31230767

